# SR-VLN: Implicit Spatial Reasoning Vision-and-Language Navigation

**DOI:** 10.3390/s26123809

**Published:** 2026-06-15

**Authors:** Ruolin Zhu, Shaobin Li, Min Yang

**Affiliations:** 1 School of Information and Communication Engineering, Communication University of China, Beijing 100024, China; 202210081000067@cuc.edu.cn; 2 School of Artificial Intelligence, Beijing University of Posts and Telecommunications, Beijing 100088, China; 2016110924@bupt.cn

**Keywords:** spatial reasoning, pyramidal hierarchical history, perceptual compression, implicit reasoning, multimodal fusion

## Abstract

Vision-and-language navigation (VLN) traditionally relies on explicit reasoning chains, which, despite being interpretable, impose severe constraints on inference efficiency and scalability in long-range environments. Existing multimodal large language models (MLLMs) frequently encounter latency bottlenecks due to the generation of verbose textual narratives during decision-making. To address these limitations, we propose spatial reasoning vision-and-language navigation (SR-VLN), a novel framework that shifts the paradigm from explicit chain-of-thought (CoT) to an implicit spatial representation space. SR-VLN introduces a pyramidal hierarchical history framework integrated with perceptual compression to condense historical trajectories into multi-scale representations, effectively minimizing token overhead while preserving critical spatial semantics. Rather than generating verbose textual reasoning steps, SR-VLN employs compact, learnable spatial tokens (S-Tokens) to perform agile inference directly within the latent feature space. To establish robust causal mappings between these implicit states and navigational actions, we employ a hybrid training strategy that combines sparse reward supervision with reinforcement learning via GRPO. Extensive evaluations on the R2R, REVERIE, and SOON datasets demonstrate that SR-VLN achieves state-of-the-art overall navigation performance, while maintaining a comparable balance between accuracy and efficiency. Compared to explicit reasoning baselines, our method reduces token consumption by 68% and achieves a 4.1× speedup in inference while reaching a 76.02% success rate and a 73.80% SPL on the R2R unseen split, thereby facilitating near-real-time action prediction in long-range navigation environments.

## 1. Introduction

Embodied artificial intelligence has seen rapid development, with vision-and-language navigation (VLN) emerging as a cornerstone challenge in robotics and multimodal learning [1,2,3]. To bridge the gap between human language and physical actions, current methods increasingly integrate multimodal large language models (MLLMs) to achieve sophisticated spatial reasoning [4,5]. While these models offer powerful cross-modal grounding, their deployment in real-time robotics is hindered by excessive computational costs and high inference latency. A significant bottleneck in current MLLM-based navigation is the reliance on explicit chain-of-thought (CoT) reasoning. In this paradigm, agents generate verbose textual justifications for their actions [5,6,7]. While enhancing interpretability, these long reasoning sequences rapidly expand in long-horizon tasks, leading to token truncation and latency issues. Additionally, explicit CoT often relies on rigid, hand-crafted templates, which limit the model’s ability to generalize to novel environment topologies. These limitations make it difficult to maintain a balance between reasoning depth and operational efficiency.

To mitigate these challenges, researchers have explored methods like adaptive routing or external memory pruning [8,9,10]. Yet, these strategies often bypass rather than resolve the core issue: the inefficiency of the reasoning process itself. Retaining full historical context in MLLMs remains computationally expensive due to redundant multimodal token sequences. Addressing this fundamental conflict requires a shift toward more compact and efficient reasoning paradigms that can handle complex multi-scale spatial layouts without the overhead of explicit text generation. In this work, we propose spatial reasoning vision-and-language navigation (SR-VLN). Our framework introduces a shift from explicit CoT to implicit spatial representations. By mapping multimodal inputs to a compact latent space, SR-VLN performs agile reasoning directly using learnable spatial tokens (S-Tokens). This approach significantly reduces the reasoning overhead while preserving rich spatial semantics. Our primary contributions are as follows:

- **Multi-scale History and Perceptual Compression.** We propose a three-tier pyramidal hierarchical history framework to organize trajectories across multiple scales, integrated with a visual autoregressive (VAR) encoding module for adaptive perceptual compression. This design condenses high-dimensional visual inputs into a compact form while maintaining structural integrity and facilitating efficient bidirectional retrieval.
- **Implicit Spatial Reasoning and Hybrid Optimization.** We introduce an implicit reasoning paradigm based on learnable S-Tokens to perform agile reasoning directly within the latent feature space. To optimize this module, we develop a hybrid training strategy integrating sparse reward supervision and GRPO, which successfully builds robust causal links between latent states and navigational actions.
- **State-of-the-Art Performance and Efficiency.** Empirical evaluations on R2R, REVERIE, and SOON benchmarks demonstrate the superiority of SR-VLN. We achieve state-of-the-art overall navigation performance while delivering a 4.1× speedup and a 68% reduction in token consumption compared to explicit reasoning methods.

## 2. Related Work

The field of vision-and-language navigation has evolved significantly since its formal introduction by Anderson et al. [1]. Early approaches relied on sequence-to-sequence architectures with attention mechanisms [2], which established the fundamental framework for mapping language instructions to navigation actions. These methods, while pioneering, struggled with generalization to unseen environments. The introduction of Transformer-based models marked a significant advancement, enabling better cross-modal alignment through self-attention mechanisms [6,11].

Recent approaches leverage pretrained vision-language models like ViLBERT [12] and LXMERT [13] for rich representation learning. These models benefit from large-scale pretraining on vision-language tasks, enabling them to capture nuanced semantic relationships. The emergence of large language models (LLMs) [14,15] has further transformed the landscape, with approaches like NaviLLM [4] and NavGPT [16] demonstrating remarkable zero-shot capabilities but suffering from substantial computational overhead. However, they primarily focus on accuracy improvements while neglecting computational efficiency. Research on physically embodied robotic platforms [17] highlights the urgent need for lightweight, 3D-aware perception solutions under tight on-device compute budgets.

### 2.1. Spatial Reasoning in VLN

Spatial reasoning [18,19] has been a central challenge in embodied AI, with researchers exploring various approaches to represent and reason about three-dimensional environments. Subsequent work by Hong et al. [20] introduced recurrent neural networks with auxiliary losses to enhance spatial reasoning capabilities. Topological mapping [21,22] represents environments as graphs where these models can capture complex dependencies between environmental elements. Methods like cognitive mapping [23,24] learn to predict spatial structure from egocentric views. GridMM [24] builds a dynamic top-down grid memory map to model global spatial relations and local visual clues. These approaches provide compact environment representations but often struggle with fine-grained spatial reasoning.

Alternative approaches have focused on learning spatial representations directly from visual observations. Explicit geometric modeling methods construct fine-grained geometric priors to enhance the spatial reasoning capability of models. GeoVLN [25] enhances visual features with geometric information from depth and normal maps, yielding more spatially grounded representations for reliable vision-language navigation. JanusVLN [18] incorporates 3D prior knowledge from the spatial-geometric encoder to strengthen the spatial reasoning capabilities of models. Despite their high accuracy, these methods are associated with high computational costs and remain too slow for real-time deployment.

### 2.2. History Modeling in VLN

In vision-language navigation (VLN), modeling historical trajectories is critical for spatial reasoning and error backtracking, as it facilitates the accumulation of environmental experience and the dynamic adjustment of navigation strategies. Early VLN endeavors include HAMT [26], the first VLN network supporting historical memory sequences and end-to-end optimization. Graph-based representations have also gained prominence, with DUET [22] introducing dual-scale graph transformers for hierarchical reasoning. To enhance temporal modeling, HOP [27,28] introduces trajectory and group order tasks to capture temporal sequence information during pre-training. However, these early approaches primarily focus on trajectory temporality while neglecting visual spatial layout in history modeling. To address this, multimodal scene memory methods [29] construct global scene memory for spatial integration. CA-VLN [30] uses MLLMs to generate historical semantic sequences fused with topological graph features. By dynamically constructing topological maps without prior experience, ETPNav [31] achieves robust navigation through a synergistic combination of a cross-modal high-level planner and an obstacle-avoiding low-level controller. MemoNav [32] proposes a tailored memory model for image-goal navigation with three collaborative memory types. Dense descriptions of historical trajectories can effectively enhance the navigation success rate of agents. Nevertheless, retrieving and reasoning about such lengthy historical information in long-range navigation inevitably incurs considerable computational overhead, which poses a critical challenge to the practical deployment of VLN systems.

## 3. Methods

### 3.1. Overall

**Figure 1:**
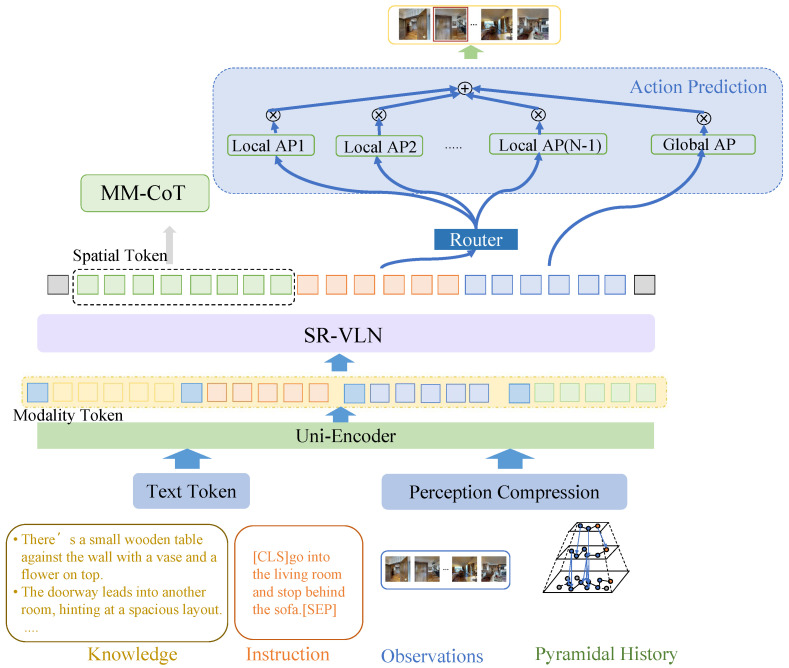
Overall architecture of SR-VLN. The framework integrates pyramidal hierarchical history memory, perceptual compression, and implicit spatial reasoning to enable efficient and robust navigation decisions. Local vs. global AP denotes local vs. global action prediction.

The overall architecture of SR-VLN is illustrated in Figure 1. The core of SR-VLN comprises three key modules: pyramidal hierarchical history, perceptual compression, and a training paradigm for implicit spatial reasoning. Given a language instruction I and a visual observation $O_{t}$, the model maintains a history $M_{1:t-1}$ of previous observations and actions. The navigation objective is to predict the next action $a_{t}$ that progresses toward the target location.

The overall framework operates in three stages. First, the perceptual compression module processes the historical context through VAR encoding, reducing token count while preserving spatial structure. Second, the pyramid history system performs bidirectional retrieval to extract relevant historical information at multiple granularities. Finally, the implicit reasoning module processes the compressed representations through learnable S-Tokens to generate action predictions.

- **MM-CoT (Multimodal Chain-of-Thought):** This is formulated as a native combination of textual CoT (T-CoT) and compressed visual CoT (CompV-CoT). The agent is required to generate paired textual–visual reasoning steps, denoted as a multimodal reasoning trace $M_{t}=\left[T_{t},V_{t}\right]$, which jointly encodes semantic plans and imagined future observations to guide subsequent action prediction.
- **Local vs. Global AP (Action Prediction):** Following the convention of topological-graph-based VLN baselines (e.g., DUET), each action predictor is implemented as a two-layer feed-forward network (FFN). To capture subtle layout differences, we increase the number of local action predictors and employ a mixture-of-experts (MoE) selection mechanism, whereas the global action predictor maintains macro-level trajectory navigation over long ranges.
- **Uni-Encoder (Unified Cross-Modal Encoder):** A multimodal backbone that serves as the central projection layer. It concurrently ingests heterogeneous inputs—including language instructions, prior spatial knowledge, visual observations, and pyramidal histories—and projects them into a shared, unified latent semantic space to facilitate seamless multimodal fusion.

### 3.2. Pyramidal Hierarchical History

**Figure 2:**
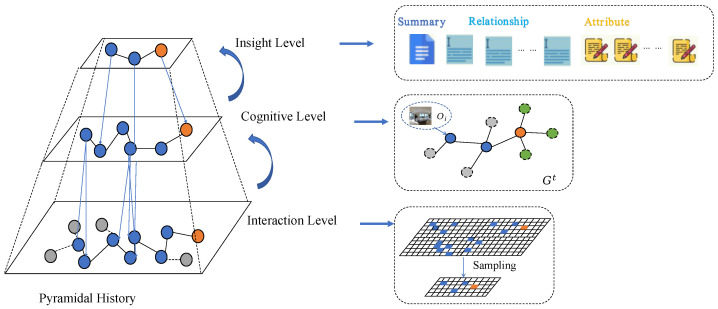
The pyramidal hierarchical history architecture. This three-tier structure organizes spatial and temporal information across the interaction, cognitive, and insight levels to enable multi-scale reasoning.

Traditional VLN models typically represent historical context through sequential lists (1D) or flat topological maps (2D), which either suffer from history overload or insufficient structural depth. We propose a three-tier pyramidal architecture that organizes trajectory information across distinct semantic scales, as depicted in Figure 2. This structure facilitates both local reactivity and global planning by segregating information based on its cognitive utility. Specifically, the pyramidal hierarchical history consists of three interconnected history levels, each serving a distinct cognitive purpose during navigation:

**Interaction Level (Short-term History)**: This level records fine-grained temporal sequences of observations and actions, $H_{\mathrm{short}}={\left\{\left(O_{i},a_{i}\right)\right\}}_{i=1}^{t-1}$, enabling the agent to perform immediate backtracking based on recent environmental feedback. Two nodes are merged if their cosine similarity exceeds $\tau_{\mathrm{cog}}=0.78$ and their geodesic distance is below δ=2.0 m. We additionally enforce a minimum cluster size m=3 and a per-episode capacity of $N_{\mathrm{cog}}^{\max}=64$ nodes. All values are obtained by grid search on R2R val-seen; sensitivity is mild (±0.4% SR within $\tau_{\mathrm{cog}}\;\in \;\left[0.74,\;0.82\right]$).

**Cognitive Level (Mid-term History)**: A topological graph $G_{\mathrm{cog}}=\left(V,E\right)$ is constructed, where nodes denote visited viewpoints and edges represent spatial connectivity. This abstraction captures the environmental topology, allowing the agent to reason about path efficiency and spatial relationships.

**Insight Level (Long-range History)**: A high-level insight graph $G_{\mathrm{insight}}=\left(N,E_{i}\right)$ stores generalized spatial motifs and transferable navigation strategies. Concretely, after each episode, we extract the induced subgraph of $G_{\mathrm{cog}}$ visited by the agent, normalize it to a canonical form (degree-ordered adjacency with discretized turning angles), and embed it using a graph neural network encoder. Recurrent motifs are discovered by cosine clustering in this embedding space, and each insight node stores the motif template together with statistics over entry/exit viewpoints and empirical success rates of local strategies. This level enables meta-reasoning across diverse environments, distilling recurring navigation patterns into actionable priors.

The construction of this pyramidal structure relies on a progressive abstraction process. At each timestep, new observations are continuously appended to the interaction level. Periodically, the system applies spatial clustering to these fine-grained memories to generate cognitive-level nodes. At the highest tier, insight-level patterns emerge through the analysis of recurring topological structures across multiple episodes, ultimately enabling transfer learning across diverse environments.

To effectively exploit this hierarchical history during active navigation, we introduce a Bidirectional Retrieval Protocol that bridges abstract strategy with grounded execution. For any given query derived from the current instruction and observation, the protocol initiates two complementary pathways. It begins with a Bottom-up Semantic Traversal, which projects query features to the insight level to retrieve relevant navigation priors:

(1) $$N^{S}=\Pi_{Q\to N}\left({\tilde{Q}}^{S}\right)$$

where ${\tilde{Q}}^{S}$ represents the expanded query set and $N^{S}$ denotes the refined semantic history. This upward projection extracts high-level navigation principles to guide the overall trajectory planning. Following this, a Top-down Contextual Traversal samples specific observations from the cognitive graph based on the retrieved insights. This downward phase enforces fine-grained spatial grounding, successfully translating abstract navigation principles into concrete actions suitable for the immediate context.

To seamlessly integrate these dual pathways, the retrieval framework employs an adaptive attention mechanism. This mechanism evaluates the relevance of different history levels to the current task, dynamically adjusting the model’s reliance on specific tiers based on environmental complexity. By structuring history access hierarchically rather than sequentially, our approach reduces retrieval computational complexity from O(n) to O(logn) while fully preserving multi-scale spatial information. We summarize the SR-VLN retrieval process in Appendix A.

### 3.3. Perceptual Compression

Visual observations contain rich spatial details but contribute significantly to token overhead. Perceptual compression employs visual autoregressive (VAR) encoding, which processes images as multi-scale pyramids while preserving spatial structure. The encoder consists of multiple resolution levels, each processing features at different scales and passing information between levels through learned gating mechanisms.

The compression process operates in two stages. First, a visual encoder extracts multi-scale features from the input image. Second, a vector quantization layer maps these features to a compact codebook representation. The decoder then reconstructs the compressed features through a series of transposed convolutions, with skip connections from the encoder preserving high-frequency details important for navigation. Concretely, the perceptual compression module adopts a multi-scale VQVAE tokenizer, which employs a shared codebook with size V=4096 across all scales, a spatial downsampling ratio of 16×, and multi-scale quantization with K additional convolution layers (adding only 0.03 M parameters). The VAR is trained using AdamW with a decay of 0.05, following the recipe of [33].

We also implement adaptive compression rates based on scene complexity. In visually rich environments with many objects, the model maintains higher fidelity compression, while in simpler scenes, more aggressive compression is applied. This adaptive mechanism ensures that computational resources are allocated efficiently across different navigation scenarios.

### 3.4. Implicit Spatial Reasoning

#### 3.4.1. Spatial Token

We define learnable implicit spatial tokens (S-Tokens) $S_{1:K}=\left(s_{1},s_{2},\ldots ,s_{K}\right)$ with fixed length *K* to encode spatial reasoning information. These tokens are modality-agnostic parameters initialized randomly and optimized to capture cross-modal spatial relationships. Each token $s_{i}\in R^{d}$ represents a learned basis vector in the reasoning space. During inference, the model composes these basis vectors through attention mechanisms to represent complex spatial relationships and navigation strategies.

The deployment of S-Tokens offers several structural advantages over conventional explicit chain-of-thought (CoT) approaches. First, by operating in a continuous latent space rather than forcing all modalities into verbose textual representations, S-Tokens inherently prevent the information loss typically encountered during cross-modal generation. Second, the fixed length *K* naturally restricts token proliferation in long-horizon tasks. While explicit CoT can generate hundreds of tokens for complex reasoning, S-Tokens guarantee a constant computational footprint regardless of task complexity. Finally, their discrete representation fundamentally avoids the error accumulation inherent to autoregressive generation, while simultaneously facilitating parallel processing and hardware-level batch optimization.

To regulate the interaction among these tokens, the S-Token architecture incorporates a learned gating mechanism. This design controls the information flow between tokens, permitting the model to selectively update specific spatial reasoning components while preserving others. Consequently, the mechanism dynamically adapts to diverse and evolving navigation scenarios.

#### 3.4.2. Training Strategy

Traditional supervised fine-tuning (SFT) enforces rigid reasoning patterns, frequently leading to overfitting. To mitigate this, we propose a three-stage training paradigm that progressively transitions from unconstrained exploration to reinforced reasoning, concluding with comprehensive knowledge distillation.

The first stage employs Sparse-Supervision Pre-training to loosen reasoning constraints. Initializing with an SFT-pretrained model, we replace explicit CoT supervision with implicit S-Token representations. In this phase, only the final action prediction receives direct supervision:

(2) $$L_{\mathrm{stage}1}=-\sum_{t=1}^{L}\log p_{\theta }\left(y_{t}|s_{<t}^{\prime }\right)$$

where $y_{t}$ is the target action and $s_{<t}^{\prime }$ represents the compressed historical context. This sparse supervision encourages the model to explore diverse internal reasoning pathways without overfitting to specific textual trajectories. An auxiliary contrastive loss is also incorporated to maintain semantically stable S-Token representations across timesteps, pulling together similar navigation contexts while pushing apart dissimilar ones.

Building upon these flexible representations, the second stage utilizes reinforcement learning to establish strict causality between S-Tokens $S_{1:K}$ and actions $y_{1:L}$. We adopt GRPO [34], defining the reward as the cosine similarity between predicted and target actions:

(3) $$r\left(y_{1:L},y^{*}\right)=\cos\left(y_{1:L},y^{*}\right)$$

Based on this reward, GRPO samples *G* trajectories $\left\{\tau_{1},\ldots ,\tau_{G}\right\}$ to compute group-relative advantages:

(4) $$J_{\mathrm{GRPO}}\left(\theta \right)=E_{\tau ∼\pi_{\theta_{\mathrm{old}}}}\left[\frac{1}{G}\sum_{i=1}^{G}\min\left(\rho_{\theta }\left(\tau_{i}\right)\hat{A}\left(\tau_{i}\right),\mathrm{CLIP}\left(\rho_{\theta }\left(\tau_{i}\right),1-\epsilon ,1+\epsilon \right)\hat{A}\left(\tau_{i}\right)\right)\right]$$

Following the standard GRPO framework, we compute relative advantages for a group of *G* sampled trajectories $\left\{\tau_{1},\ldots ,\tau_{G}\right\}$ without a critic network:

(5) $$\hat{A}\left(\tau_{i}\right)=\frac{R\left(\tau_{i}\right)-\mathrm{mean}\left(\left\{R\left(\tau_{1}\right),\ldots ,R\left(\tau_{G}\right)\right\}\right)}{\mathrm{std}(\left\{R\left(\tau_{1}\right),\ldots ,R\left(\tau_{G}\right)\right\})+\epsilon }$$

where $R(\tau_{i})$ is the cumulative return of trajectory $\tau_{i}$, and $\epsilon =10^{-8}$. A trajectory is parameterized as $\tau =\{s_{1},S_{1},y_{1},\ldots ,s_{T},S_{T},y_{T}\}$, constrained by a maximum rollout horizon of H=15 steps. The total return R(τ) combines the step-wise dense cosine reward and the sparse terminal success reward:

(6) $$R\left(\tau \right)=\frac{\lambda_{1}}{T}\sum_{t=1}^{T}r\left(y_{t},y_{t}^{*}\right)+\lambda_{2}\cdot I_{\mathrm{success}}$$

where $I_{\mathrm{success}}\in \left\{0,1\right\}$ indicates whether the agent successfully stops within 3 m of the target, and we set $\lambda_{1}=0.5$, $\lambda_{2}=1.0$ to balance step-by-step guidance and global objective completion.

This gradient signal propagates directly through the S-Tokens, reinforcing trajectories with rigorous spatial computation while penalizing shortcut paths. A curiosity-driven exploration bonus is further integrated to prevent premature convergence to suboptimal reasoning patterns. Combining these first two stages ensures the model simultaneously learns useful latent representations and optimizes them for accurate navigation, avoiding the pitfalls of skipping either exploration or reinforcement.

The final stage leverages knowledge distillation to transfer reasoning capabilities from a high-capacity teacher model (Qwen2.5-VL-32B) to the target student model (Qwen2.5-VL-7B). This process compensates for potential information loss during history compression. The core distillation loss aligns the logits of the S-Token:

(7) $$L_{\mathrm{distill}}=\left|\right|P_{t}^{T}-P_{t}{\left|\right|}_{2}^{2}$$

where $P_{t}^{T}$ and $P_{t}$ denote the teacher and student tokens, respectively. Beyond basic representation matching, this stage implements a progressive, curriculum-based distillation that aligns both intermediate features and attention patterns, ensuring the student accurately replicates the teacher’s spatial focus. The complete student objective combines this distillation with direct action prediction:

(8) $$L_{\mathrm{student}}=L_{\mathrm{distill}}-\sum_{t=1}^{L}\log p_{\theta }\left(y_{t}|s_{<t}^{\prime }\right)$$

Supported by a supplementary consistency loss on unlabeled episodes, this semi-supervised approach leverages the teacher’s generalization capabilities to produce a highly robust student model capable of excelling in out-of-distribution scenarios.

## 4. Experimental

### 4.1. Datasets

We evaluate on three standard VLN benchmarks that span different difficulty levels and task requirements: R2R [1] contains 7189 navigation paths with 21,567 instructions across 90 environments. This dataset focuses on room-to-room navigation with relatively short trajectories and clear language instructions. REVERIE [35] focuses on remote object grounding with 21,702 instructions. This dataset requires agents to find specific objects in complex environments, testing high-level semantic understanding and long-horizon planning. SOON [36] requires finding object instances in complex scenes with 40,541 instructions. This dataset presents the greatest challenge, with ambiguous instructions and complex multi-room environments.

### 4.2. Evaluation Metrics

All three datasets are based on the Matterport3D [37] environment and simulate dynamic and noisy scenarios. Following the evaluation protocol implemented on the open-source platform EvalAI [38], we report five key metrics for comprehensive performance assessment. Trajectory Length (TL) refers to the average path length measured in meters, which quantifies navigation efficiency. Navigation Error (NE) represents the Euclidean distance between the agent’s final stopping position and the target goal location, which evaluates navigation accuracy. Success Rate (SR) is defined as the percentage of navigation episodes where the NE value is below 3 m, which characterizes task completion capability. Success weighted by Path Length (SPL) weights successful navigation cases by the ratio of optimal shortest path length to the agent’s actual path length, jointly measuring success and path efficiency. Time per action (TPA) refers to the average time consumed to execute a single navigation action, which quantifies the inference efficiency of the algorithm.

### 4.3. Implementation Details

During the continuous pre-training stage of the large model, training is conducted on 8 H100 GPUs. The AdamW optimizer is adopted with a dropout rate of 0.05, and the learning rate is initialized as $10^{-5}$. The learning rate warm-up procedure is set to 800 steps, followed by cosine annealing. The per-GPU batch size is set to 8, and the total number of training iterations is 100 k. The hidden feature dimension is configured as 768, and the maximum input sequence length is set to 2048 to satisfy the processing requirements of multimodal image-text input data. The hyperparameter *K*, representing the implicit visual feature reasoning length, is set to 5.

In the sparse training stage, the batch size is set to 4. A maximum gradient norm of 5 is enforced, and the bfloat16 floating-point precision is utilized, with a learning rate of $10^{-6}$. The total number of training iterations is 2500. For the GRPO training stage, the KL divergence weight β is set to 0.04. To ensure training stability, we apply a weight decay rate of 0.01 and clip the maximum gradient norm to ${∥\nabla \theta ∥}_{2}\leq 1.0$. The maximum response length is set to 768 tokens, and the learning rate is initialized as 1 $\times 10^{-6}$. A hybrid dataset is employed across all training stages, and other hyperparameters follow the standard settings [39,40].

### 4.4. Comparison with State-of-the-Art

Table 1 presents comprehensive results on the R2R validation set. Our SR-VLN achieves 82.61% SR on seen environments and 76.02% on unseen environments, substantially outperforming all previous baselines. Compared to DUET, which achieves 71.52% SR on unseen scenes, SR-VLN provides a 4.5% absolute improvement. More impressively, SR-VLN improves SPL by 13.8% on unseen scenes compared to DUET (73.80% vs. 60.00%), demonstrating significantly more efficient path planning.

The performance gap is even more pronounced when compared to earlier methods like Seq2seq (22.00% SR) and RCM (43.00% SR), highlighting the substantial progress made by recent approaches. SR-VLN also outperforms other large model-based approaches like NaviLLM (67.00% SR) and NavCot (40.00% SR), demonstrating the effectiveness of our efficiency-focused design.

On the R2R test set, which provides the most rigorous evaluation of generalization, SR-VLN achieves 73.00% SR on unseen environments, surpassing DUET (69.25%) and NaviLLM (68.00%). NE is reduced to 3.36, indicating more accurate target localization. These results demonstrate that SR-VLN maintains strong performance even on the most challenging evaluation split.

Table 2 shows results on the REVERIE dataset, which tests object grounding capabilities. SR-VLN achieves 59.41% SR on the test unseen split, outperforming DUET (52.42%) by a substantial 6.99% margin. This improvement is particularly significant as REVERIE requires precise object localization in addition to navigation.

Table 3 presents results on the SOON dataset, which presents the greatest challenge due to ambiguous instructions and complex multi-room environments. SR-VLN achieves 26.31% SPL on the test unseen split, with an RGSPL of 5.92%. While the absolute numbers are lower than on R2R and REVERIE due to the dataset’s difficulty, SR-VLN still outperforms baselines on path efficiency metrics.

In summary, the comparative analysis with GOAT reveals clear performance trade-offs that are deeply rooted in the distinct characteristics of different navigation paradigms. On the one hand, our SR-VLN consistently maintains a clear advantage over GOAT in terms of success rate weighted by path length (SPL), which directly demonstrates that our implicit spatial reasoning framework is inherently more proficient at global spatial planning to yield more optimal paths. On the other hand, while GOAT exhibits slightly better overall metrics on the fine-grained, instruction-heavy R2R dataset, SR-VLN achieves superior performance on goal-oriented datasets such as REVERIE and SOON. This task-specific variation is tightly coupled with the respective objectives: the R2R dataset relies heavily on detailed, long step-by-step sequential descriptions, whereas REVERIE and SOON demand higher-level object-grounding and macroscopic spatial inference. The remarkable results on these goal-oriented benchmarks further validate that the implicit spatial tokens (S-Tokens) in SR-VLN endow the agent with stronger macro-level spatial reasoning capabilities, which are crucial for executing complex, long-range exploration toward specified targets.

### 4.5. Ablation Studies

We conduct comprehensive ablation studies to validate each component’s contribution. Table 4 shows the incremental benefits of adding VAR compression and S-Tokens to the base UniVLN model. The base model achieves 79.94% SR on seen environments. Adding VAR compression alone improves SR to 80.23% while reducing tokens by 68%, demonstrating that compression does not harm performance. Incorporating S-Tokens further boosts SR to 80.51% with superior SPL (81.2%), showing that implicit reasoning provides additional benefits. The complete SR-VLN model achieves the best performance (82.61% SR, 82.4% SPL), demonstrating complementary benefits of compression and implicit reasoning.

#### 4.5.1. Efficiency Analysis

Table 5 presents detailed efficiency comparisons. SR-VLN achieves 0.93 TPA, representing a 4.1× speedup over the explicit reasoning baseline model (3.84 TPA). This improvement comes primarily from two sources: (1) VAR compression reduces visual tokens by 68%, cutting computation in the vision encoder, and (2) S-Tokens replace lengthy explicit reasoning sequences with fixed-length representations.

We also measure the breakdown of inference time. For SR-VLN, visual encoding takes 45% of total time, S-Token processing takes 30%, and action prediction takes 25%. In contrast, UniVLN spends 60% of its time on visual encoding and 35% on reasoning generation, demonstrating the efficiency gains from our approach.

#### 4.5.2. Training Paradigm Analysis

Table 6 validates our two-stage training strategy. Using only sparse-supervision pre-training degrades performance (74.37% SR) due to a lack of reasoning supervision. Pure reinforcement learning also underperforms (72.45% SR) because models trained solely with RL tend to overfit to reward signals without developing robust internal representations. Our combined approach achieves the best performance (81.6% SR), confirming that sparse rewards enable exploration while RL provides necessary constraints.

We also analyze the effect of different RL algorithms. PPO achieves 79.2% SR, while our GRPO variant achieves 81.6% SR, demonstrating the benefits of group-relative advantage estimation. The group size of 8 provides optimal performance, balancing variance reduction with computational overhead.

#### 4.5.3. Knowledge Distillation Effectiveness

Distillation from Qwen2.5-VL-32B to Qwen2.5-VL-7B significantly improves performance. Table 7 shows that the distilled 7B model achieves 82.61% SR, approaching the teacher’s 82.81% while maintaining inference efficiency. This confirms that our distillation method effectively transfers spatial reasoning capabilities.

We also compare different distillation strategies. Logit distillation alone achieves 81.9% SR, while our feature-based approach achieves 82.61% SR. Adding attention distillation provides an additional 0.2% improvement, confirming the value of aligning internal representations.

#### 4.5.4. Impact of S-Token Length *K*

**Figure 3:**
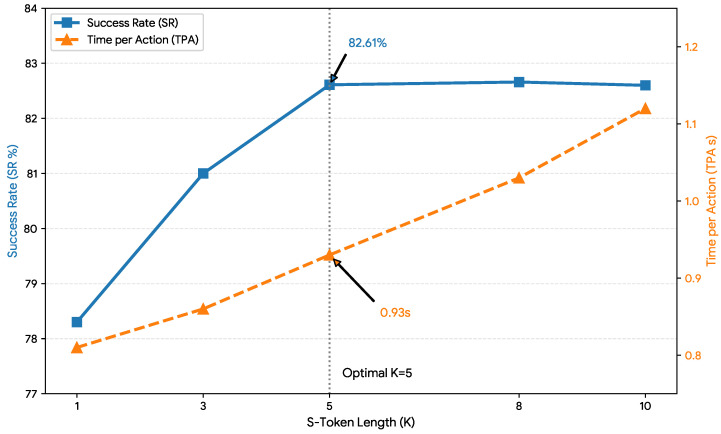
Trade-off between S-Token length *K* and navigation performance/efficiency.

Figure 3 analyzes the effect of S-Token length *K* on performance and efficiency. As *K* increases from 1 to 10, SR improves from 78.3% to 82.6%, but TPA increases from 0.84 to 1.15. K=5 provides the optimal balance, achieving 82.61% SR with 0.93 TPA. This demonstrates that a compact implicit representation suffices for effective spatial reasoning.

### 4.6. Qualitative Analysis

**Figure 4:**
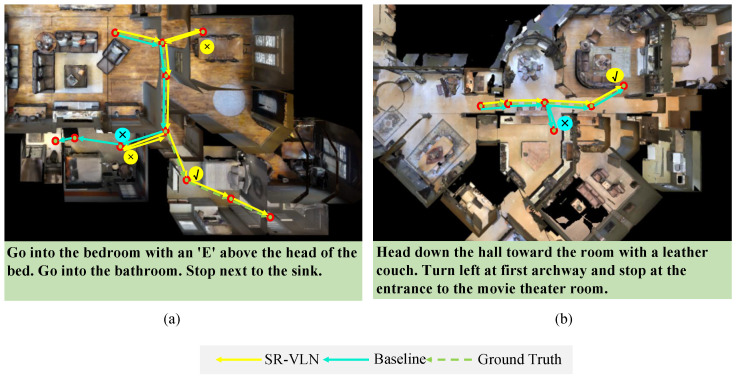
Qualitative trajectory comparison. SR-VLN (yellow) demonstrates more efficient path planning than the baseline (cyan) in a multi-room navigation scenario. Subfigures (**a**,**b**) illustrate examples in two different cases.

Figure 4 provides a qualitative visualization of navigation trajectories in challenging scenarios, illustrating how our model effectively bridges three prominent text-visual alignment gaps. The first challenge stems from unmapped environments with repetitive functional spaces (e.g., distinguishing the target bedroom from multiple similar candidate rooms). The second lies in identifying fine-grained semantic text cues, such as aligning the alphanumeric token “E” with its local spatial location (“above the head of the bed”) in Figure 4a, or discerning a “leather couch” and a “movie theater room” in Figure 4b. The third challenge is the total absence of explicit intermediate path descriptions within the textual instructions, which deprives the agent of step-by-step guidance and forces it to rely entirely on independent, global spatial exploration.

Compared to DUET, which frequently suffers from fine-grained semantic recognition errors and misses potential pathways, SR-VLN demonstrates a superior capability to balance global exploration with fine-grained local text-visual comprehension. As illustrated in the sequence of Figure 4a, while standard navigation models fail to accurately align the delicate constraint “bedroom with an ’E”’ and consequently misidentify the relative position of the final goal, SR-VLN systematically explores the sequentially distributed adjacent rooms. Although the layout ambiguity causes our model to temporarily detour into an incorrect room, its implicit reasoning mechanism immediately detects the visual-textual mismatch (i.e., the absence of the letter “E” in the current scene). This triggers an agile, autonomous self-correction, enabling the agent to promptly leave the wrong scene and successfully locate the correct destination next to the sink in the bathroom.

A similar superiority is observed in Figure 4b. Faced with the sparse instruction “Head down the hall…”, the baseline model completely overlooks the adjacent rooms visible from its current viewpoint, failing to properly ground the landmarks. In contrast, SR-VLN maintains an exhaustive spatial awareness without leaving any viewpoint unvisited. It accurately aligns the landmark cues (“leather couch” and “first archway”) to successfully navigate the long-range, unguided trajectory toward the entrance of the movie theater room. This adaptive error-correction and comprehensive environmental tracking confirm that SR-VLN can robustly handle high-ambiguity instructions and complex spatial layouts.

We also analyze failure cases. SR-VLN tends to struggle in scenarios with extremely ambiguous instructions or when the target object is occluded. In such cases, the model sometimes overconfidently commits to incorrect paths. Future work could incorporate uncertainty estimation to enable more cautious decision-making in ambiguous situations.

## 5. Conclusions

This work proposes and validates SR-VLN, a framework accelerated through implicit spatial reasoning. By integrating pyramidal hierarchical history management, context-aware perceptual compression, and a novel training paradigm for implicit spatial reasoning, SR-VLN effectively addresses the challenges faced by existing VLN methods in long-range navigation, inference efficiency, and generalization. Extensive experiments on multiple standard datasets show that SR-VLN achieves state-of-the-art performance in terms of both success rate and path efficiency, with particularly strong results in unseen environments. These findings provide new insights and a solid technical foundation for building more efficient and robust navigation systems in embodied intelligence and constitute a step toward deploying general-purpose navigation agents in the real world.

## Figures and Tables

**Table 1:** Performance Comparison on the R2R dataset.

Models	Val_Seen				Val_Unseen				Test_Unseen			
	TL ↓	NE ↓	SR ↑	SPL ↑	TL ↓	NE ↓	SR ↑	SPL ↑	TL ↓	NE ↓	SR ↑	SPL ↑
Seq2seq [1]	11.33	6.01	39	-	8.29	7.81	22	18	**8.13**	7.85	20	18
RCM [41]	10.65	3.53	67	-	11.46	6.09	43	-	9.48	4.21	60.5	59
*Pre-trained VLN Algorithms*												
VLN-BERT [20]	11.13	2.90	72	68	12.01	3.93	63	57	12.35	4.09	63	57
AirBERT [42]	11.09	2.68	74.85	69	10.03	3.24	68.67	63	-	-	-	-
HAMT [26]	11.15	2.51	76	72	11.46	3.62	66	61	12.27	3.93	65	60
Lily [43]	10.21	2.89	79.31	76	10.03	3.19	70	65	-	-	-	-
HOP+ [28]	11.31	2.33	78	73	11.76	3.49	67	61	12.67	3.71	66	60
ESceme [29]	10.65	2.57	76	73	10.80	3.39	68	64	11.89	3.77	66	63
KERM [44]	12.16	2.19	80	74	13.54	3.22	72	61	14.6	3.61	70	59
ACME [45]	11.28	2.16	80.12	75.68	12.23	3.12	72.75	62.3	13.43	3.68	70.4	61.2
GOAT [46]	-	1.79	83.74	79.48	-	2.40	77.82	68.13	-	3.04	74.57	64.94
DUET [22]	12.31	2.28	78.84	72.89	13.94	3.31	71.52	60	14.74	3.65	69.25	58.68
*Large Model-based VLN*												
NaviLLM [4]	-	-	-	-	-	-	-	-	13.21	3.71	68	60
NavCot [7]	10.08	6.46	41	38	9.95	6.26	40	37	-	-	-	-
EvolveNav [47]	-	-	-	-	13.43	3.27	70.11	60.25	13.68	3.37	70	61
CA-VLN [30]	11.84	2.04	81.03	75.15	12.70	3.03	73.31	61.95	14.41	3.37	70.27	60.31
SR-VLN (Ours)	11.16	1.94	82.61	**82.4**	10.6	3.07	76.02	**73.8**	13.07	3.36	73	62.4

**Table 2:** Performance Comparison of the REVERIE dataset.

Models	Val_Seen			Val_Unseen			Test_Unseen		
	OSR ↑	SR ↑	SPL ↑	OSR ↑	SR ↑	SPL ↑	OSR ↑	SR ↑	SPL ↑
Seq2seq [1]	35.7	29.59	24.01	8.07	4.2	2.84	6.88	3.99	3.09
RCM [41]	29.44	23.33	21.82	14.23	9.29	6.97	11.68	7.84	6.67
*Pre-trained VLN Algorithms*									
VLN-BERT [20]	-	-	-	35.02	30.67	24.9	32.91	29.61	23.99
Lily [43]	-	-	-	53.71	48.11	34.43	60.51	54.32	37.34
HOP+ [28]	72.37	69.34	63.64	54.02	49.51	36.3	59.34	54.28	38.64
ESceme [29]	73.14	70.47	64.12	54.37	50.42	36.41	59.47	55.31	39.27
KERM [44]	74.49	71.89	64.04	52.21	50.44	35.38	57.58	52.43	39.21
ACME [45]	74.94	71.96	64.45	53.97	49.46	32.27	57.48	51.89	34.65
AZHP [48]	75.12	74.14	67.22	53.65	48.31	36.63	55.31	51.57	35.85
GOAT [46]	-	78.64	71.40	-	53.37	36.70	-	57.72	40.53
DUET [22]	72.59	70.34	62.96	51.12	46.46	33.18	56.87	52.42	35.98
*Large Model-based VLN*									
NaviLLM [4]	-	-	-	53.74	44.56	36.63	56.21	43.49	34.45
NavCot [7]	-	-	-	44.28	39.64	29.3	46.32	40.15	30.14
EvolveNav [47]	-	-	-	42.40	33.60	28.16	-	-	-
CA-VLN [47]	74.94	72.37	63.67	53.94	49.37	35.67	60.73	56.41	36.48
SR-VLN (Ours)	**77.36**	75.93	65.31	**55.2**	49.61	**37.63**	**62.41**	**59.41**	**41.38**

**Table 3:** Performance Comparison of the SOON dataset.

Models	Val_Unseen				Test_Unseen			
	OSR ↑	SR ↑	SPL ↑	RGSPL ↑	OSR ↑	SR ↑	SPL ↑	RGSPL ↑
GBE [36]	28.54	19.52	13.34	1.16	21.45	12.90	9.23	0.45
KERM [44]	51.62	38.05	23.16	-	-	-	-	-
ACME [45]	50.73	38.43	27.81	3.73	43.51	35.55	22.28	4.11
AZHP [48]	-	72	61	-	-	22.28	4.11	-
GOAT [46]	54.69	40.35	28.05	5.85	50.63	40.50	25.18	6.10
DUET [22]	51.01	36.30	22.37	4.04	42.95	33.23	21.15	5.47
NaviLLM [4]	33.11	19.81	14.29	4.33	-	-	-	-
CA-VLN [30]	54.13	37.32	21.90	3.60	44.87	34.35	22.64	5.26
EvolveNav [47]	49.56	33.40	24.92	-	-	-	-	-
SR-VLN (Ours)	54.34	**41.16**	**29.01**	4.89	47.28	35.16	**26.31**	5.92

**Table 4:** Ablation study of SR-VLN components on the R2R validation set.

Configuration	Seen			Unseen		
	TL ↓	SR ↑	SPL ↑	TL ↓	SR ↑	SPL ↑
Base (w/o S-Tokens and PHH)	10.87	79.94	81.00	11.60	74.68	73.00
Base + PHH	12.36	80.23	80.70	12.42	74.80	73.20
Base + S-Tokens	11.64	80.51	81.20	10.30	75.03	73.60
SR-VLN (Full)	11.16	82.61	82.40	10.60	76.02	73.80

**Table 5:** Inference efficiency comparison.

Model	Reasoning Type	TPA
CA-VLN [30]	Explicit	7.14
SR-VLN w/o S-Tokens and PHH	Explicit	3.84
Aux-Think [49]	Implicit	1.03
SR-VLN	Implicit	**0.93**

**Table 6:** Ablation of training strategies on the R2R validation set.

Training Strategy	TL ↓	NE ↓	SR ↑	SPL ↑
SFT Pretrained	12.36	2.05	80.23	80.70
Only Sparse Reward	13.94	3.24	74.37	78.90
Only RL (GRPO)	14.63	3.72	72.45	76.30
Two-Stage (Ours)	11.94	2.17	81.60	81.80

**Table 7:** Knowledge distillation results on the R2R validation set.

Model	TL ↓	NE ↓	SR ↑	SPL ↑
Undistilled 7B	11.94	2.17	81.60	81.80
Teacher (32B)	10.13	1.90	82.81	82.90
Distilled 7B	11.16	1.94	82.61	82.40

## Data Availability

The datasets used in this manuscript are publicly available datasets. Detailed information about these datasets is provided in Section 4.1.

## Abbreviations

The following abbreviations are used in this manuscript:

VLN	Visual-Language Navigation
VAR	Autoregressive
PHH	Pyramidal Hierarchical History
CoT	Chain-of-Thought

## Appendix A. Retrieval Process of SR-VLN

**Algorithm A1** Pyramidal hierarchical history retrieval
1: **Input:** Current query *q*, Query graph $G_{\mathrm{query}}$, Insight graph $G_{\mathrm{insight}}$, Interaction graph $G_{\mathrm{interact}}$ 2: **Output:** Semantic history $N^{S}$, Contextual history $H^{C}$ 3: // Bottom-up traversal 4: Find the relevant query set ${\tilde{Q}}^{S}$$\subseteq G_{\mathrm{query}}$ related to *q*. 5: $N^{S}\leftarrow \emptyset$ 6: **for** each insight node $n_{k}\in G_{\mathrm{insight}}$ **do** 7: **if** support_query_set($n_{k}$) ∩${\tilde{Q}}^{S}$≠∅ **then** 8: $N^{S}\leftarrow N^{S}\cup \left\{n_{k}\right\}$ 9: **end if** 10: **end for** 11: // Top-down traversal 12: $H^{C}\leftarrow \emptyset$ 13: Retrieve high-level strategy from $G_{\mathrm{insight}}$ based on *q*. 14: Sample relevant observation points from $G_{\mathrm{interact}}$ based on the strategy. 15: $H^{C}\leftarrow$ features of sampled points. 16: **return** $N^{S},H^{C}$

## Appendix B. Computational Complexity Analysis

This work introduces a suite of inference acceleration techniques for the large navigation model, preserving the original computational complexity while substantially boosting inference efficiency. For the 7B-parameter model, inference latency is reduced to $\tilde{9}$30 ms on a single NVIDIA Tesla H100 GPU, with an additional $\tilde{1}$2 ms overhead from the retrieval module. The TPA is 0.93, with average per-inference latency at the second-scale level. The history bank-based sampling strategy also fixes the number of historical feature tokens, decoupling inference efficiency from the navigation path length. Perceptual compression shrinks the per-frame visual feature to $\tilde{3}$2% of its original size, drastically reducing history consumption for historical visual representations. The history overhead of historical textual sequences is negligible compared to that of visual features and is easier to maintain.

## Appendix C. Hyperparameters of the Pyramidal Hierarchical History

**Table A1:** Hyperparameters of the pyramidal hierarchical history.

Symbol	Meaning	Value
$\tau_{\mathrm{cog}}$	Cosine merge threshold (interaction → cognitive)	0.85
δ	Max geodesic distance for merge (m)	2.0
*m*	Minimum cluster size	3
$N_{\mathrm{cog}}^{\max}$	Max cognitive-level nodes per episode	64
$N_{\mathrm{ins}}$	Insight-graph bound (cross-episode)	16
$K_{\mathrm{merge}}$	Async re-merge cadence (steps)	8

## Appendix D. GRPO Group-Size Sensitivity

We sweep G∈{4,8,12,16} and report the trade-off between final SR and per-step wall-clock cost in Table A2. The advantage-estimator variance behaves as ∝1/(G−1), as predicted by the GRPO derivation; gains saturate beyond G=8 (ΔSR<0.2% for G≥12), while training time grows nearly linearly. G=8 therefore sits on the Pareto knee and is retained as the default.

**Table A2:** GRPO group-size sensitivity.

*G*	SR ↑	SPL ↑	Train Wall-Clock (Rel.) ↓
4	74.8	72.80	0.72
8	76.02	73.80	1.00
12	76.10	73.85	1.45
16	76.18	73.90	1.91
